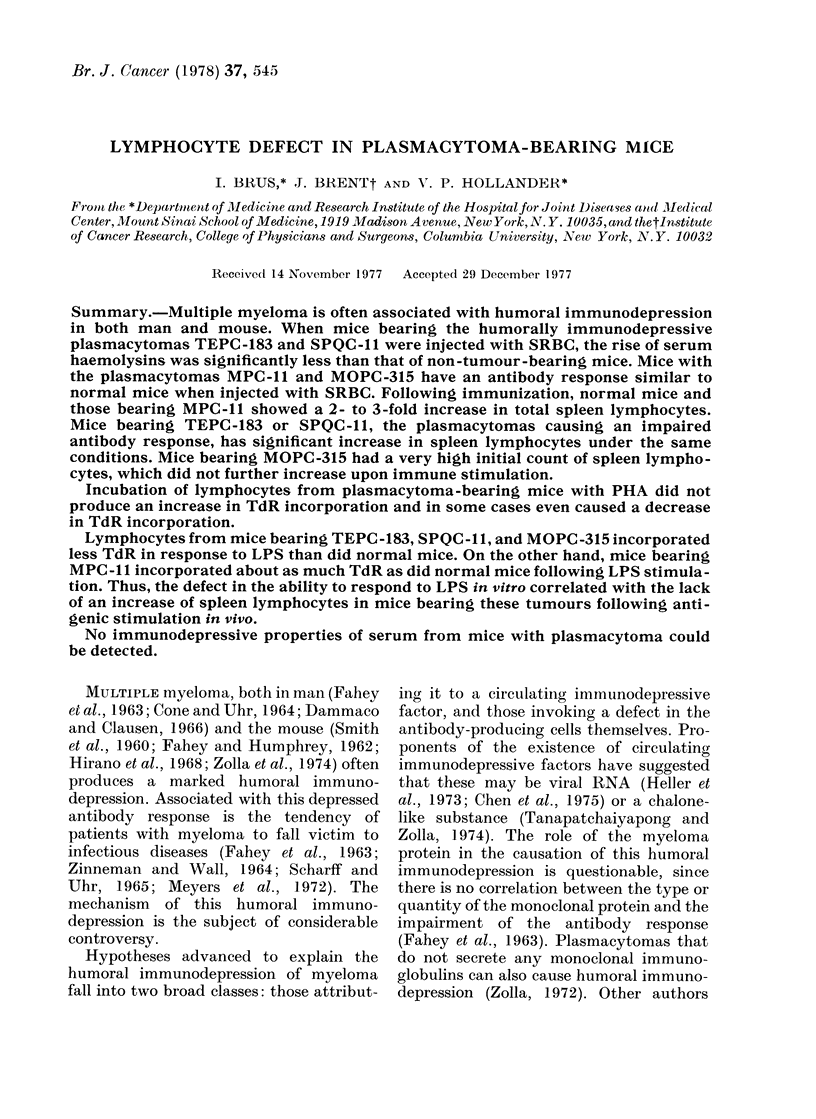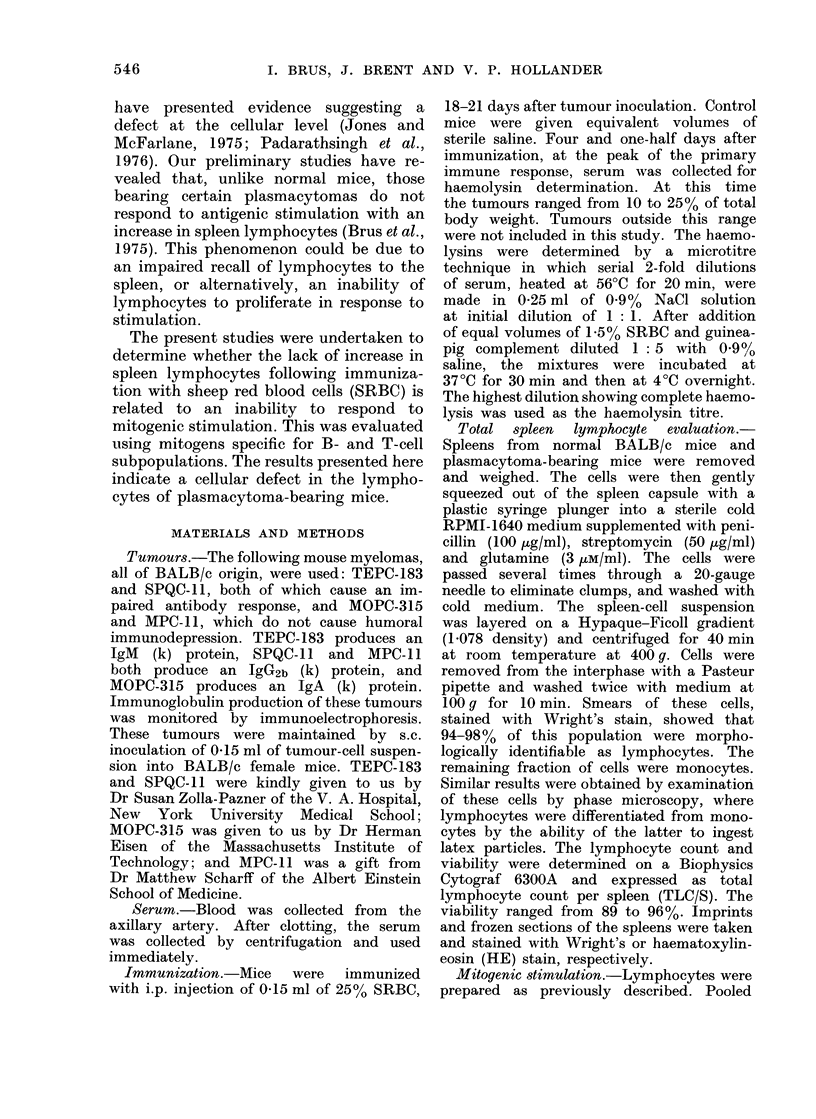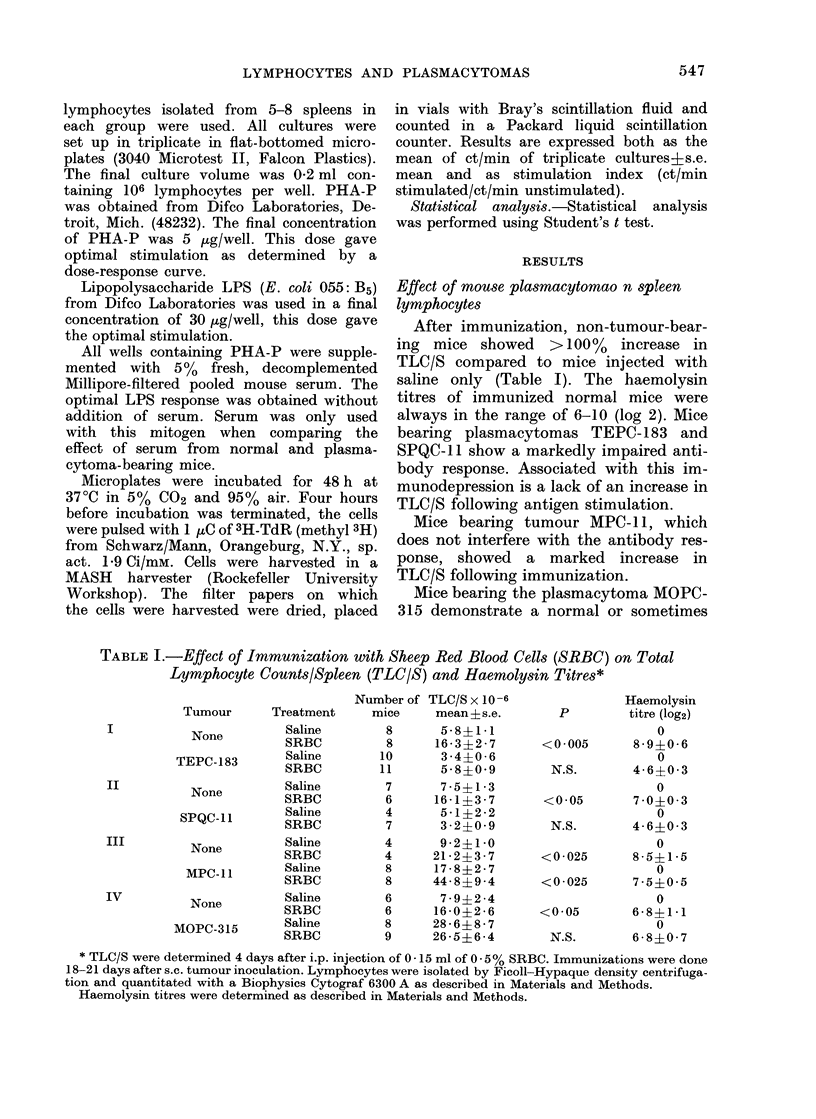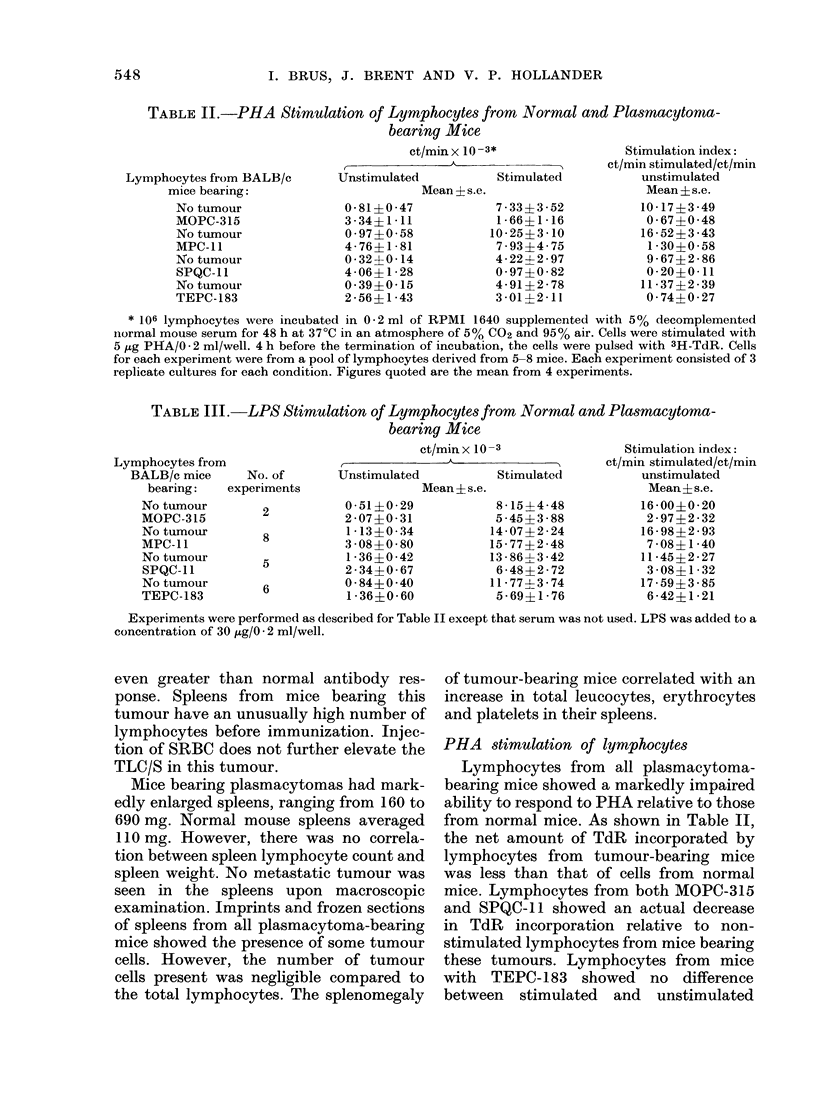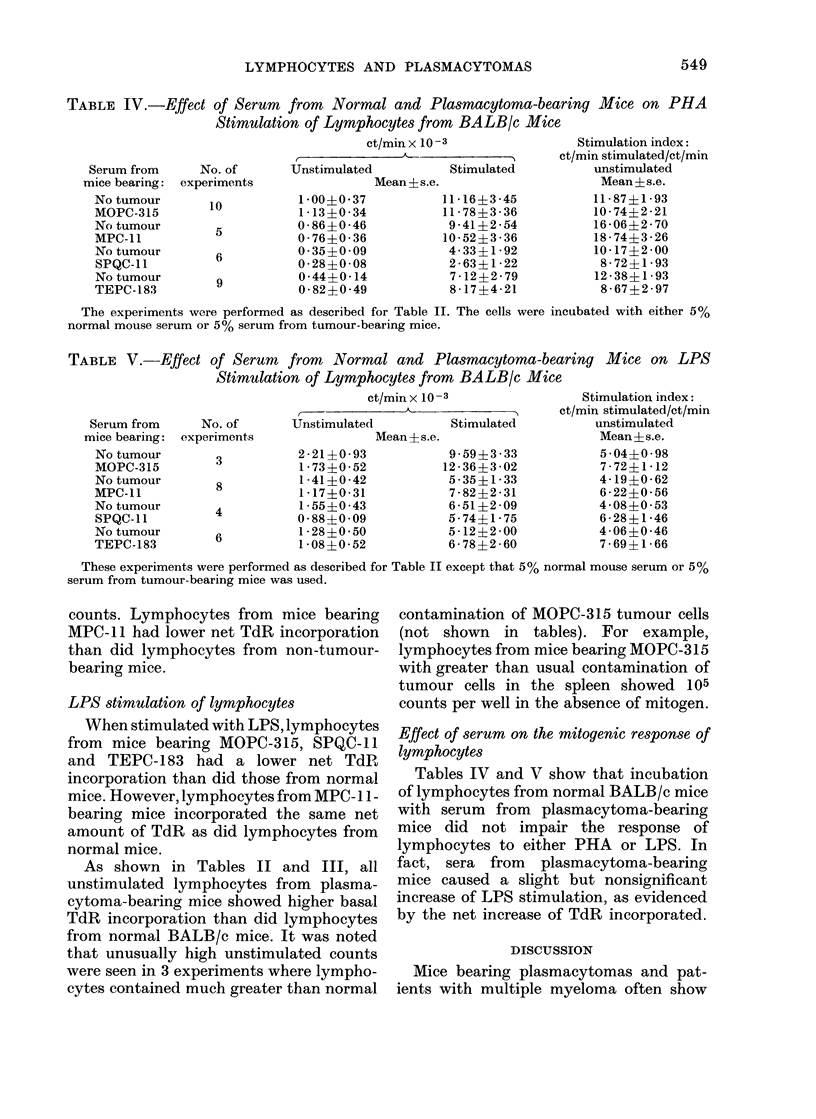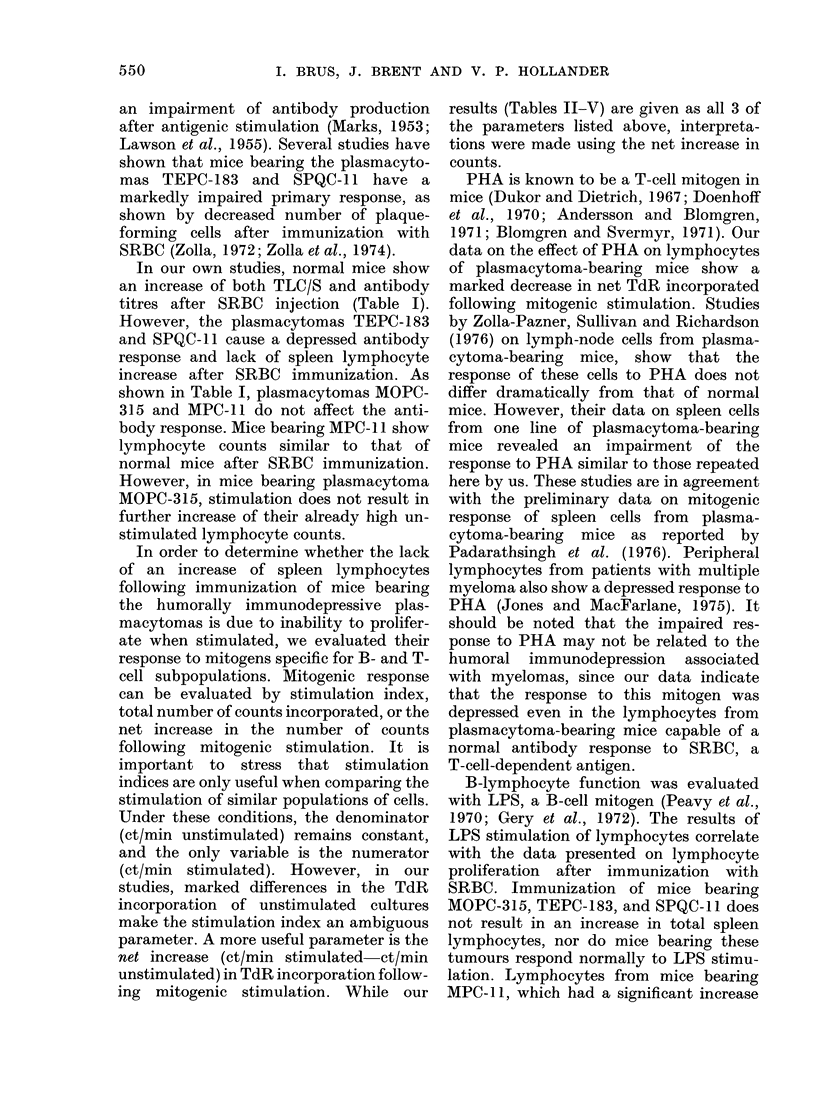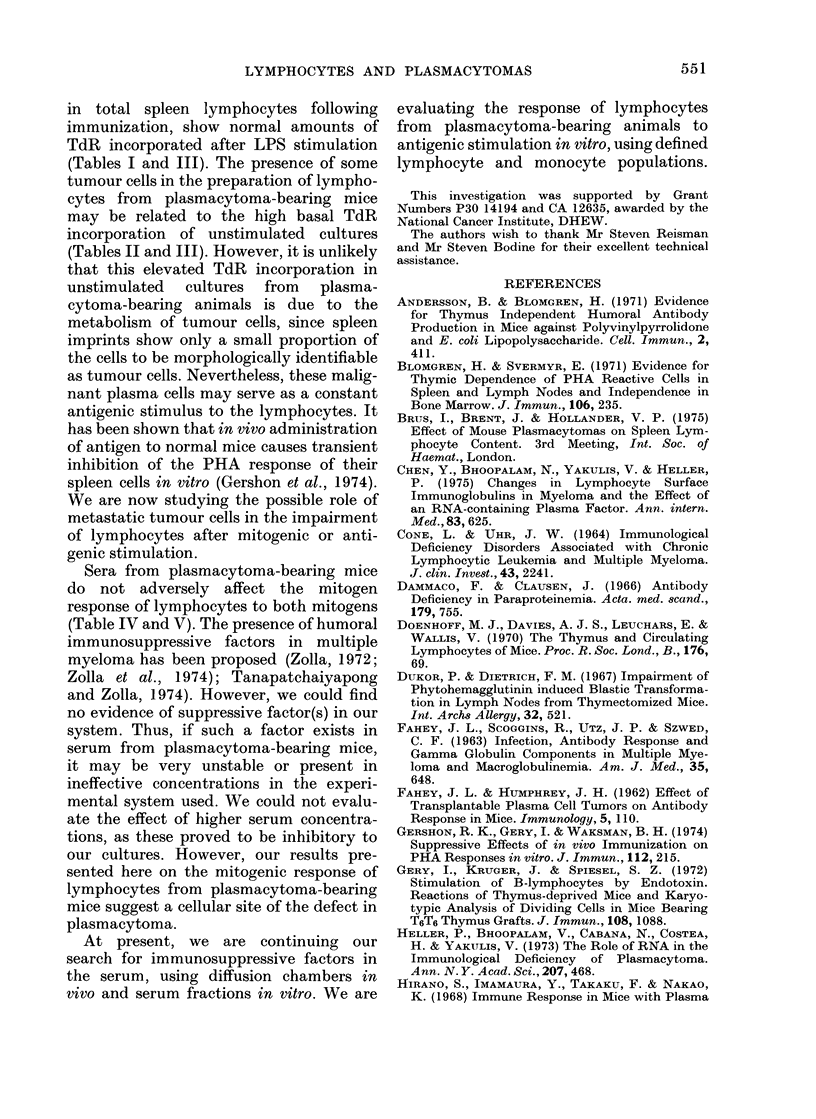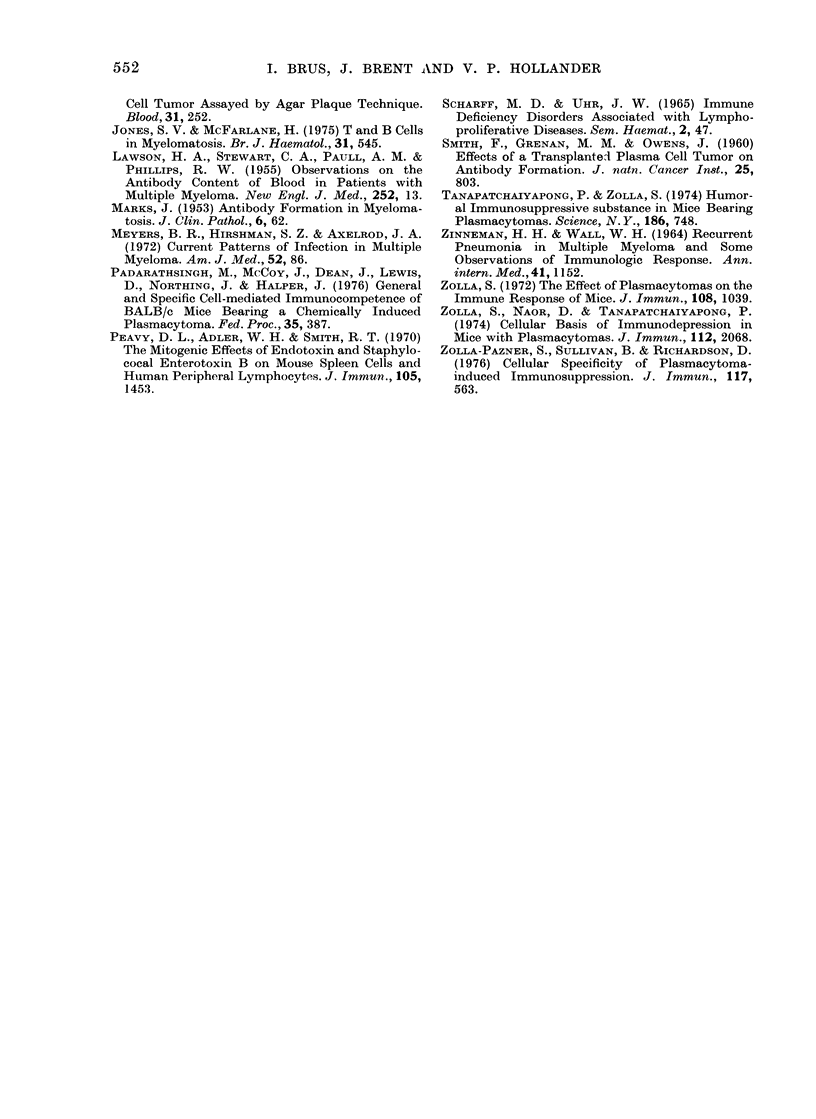# Lymphocyte Defect in Plasmacytoma-bearing Mice

**DOI:** 10.1038/bjc.1978.83

**Published:** 1978-04

**Authors:** I. Brus, J. Brent, V. P. Hollander

## Abstract

Multiple myeloma is often associated with humoral immunodepression in both man and mouse. When mice bearing the humorally immunodepressive plasmacytomas TEPC-183 and SPQC-11 were injected with SRBC, the rise of serum haemolysins was significantly less than that of non-tumour-bearing mice. Mice with the plasmacytomas MPC-11 and MOPC-315 have an antibody response similar to normal mice when injected with SRBC. Following immunization, normal mice and those bearing MPC-11 showed a 2- to 3-fold increase in total spleen lymphocytes. Mice bearing TEPC-183 or SPQC-11, the plasmacytomas causing an impaired antibody response, has significant increase in spleen lymphocytes under the same conditions. Mice bearing MOPC-315 had a very high initial count of spleen lymphocytes, which did not further increase upon immune stimulation.

Incubation of lymphocytes from plasmacytoma-bearing mice with PHA did not produce an increase in TdR incorporation and in some cases even caused a decrease in TdR incorporation.

Lymphocytes from mice bearing TEPC-183, SPQC-11, and MOPC-315 incorporated less TdR in response to LPS than did normal mice. On the other hand, mice bearing MPC-11 incorporated about as much TdR as did normal mice following LPS stimulation. Thus, the defect in the ability to respond to LPS *in vitro* correlated with the lack of an increase of spleen lymphocytes in mice bearing these tumours following antigenic stimulation *in vivo*.

No immunodepressive properties of serum from mice with plasmacytoma could be detected.


*Br. J. Cancer* (1978) **37**, 545

### LYMPHOCYTE DEFECT IN PLASMACYTOMA-BEARING MICE

I. BRUS,* J. BRENT† AND V. P. HOLLANDER*

*From the *Department of Medicine and Research Institute of the Hospital for Joint Diseases and Medical Center, Mount Sinai School of Medicine, 1919 Madison Avenue, New York, N.Y. 10035, and the †Institute of Cancer Research, College of Physicians and Surgeons, Columbia University, New York, N.Y. 10032*

Received 14 November 1977 Accepted 29 December 1977

**Summary.—Multiple myeloma is often associated with humoral immunodepression in both man and mouse. When mice bearing the humorally immunodepressive plasmacytomas TEPC-183 and SPQC-11 were injected with SRBC, the rise of serum haemolysins was significantly less than that of non-tumour-bearing mice. Mice with the plasmacytomas MPC-11 and MOPC-315 have an antibody response similar to normal mice when injected with SRBC. Following immunization, normal mice and those bearing MPC-11 showed a 2- to 3-fold increase in total spleen lymphocytes. Mice bearing TEPC-183 or SPQC-11, the plasmacytomas causing an impaired antibody response, has significant increase in spleen lymphocytes under the same conditions. Mice bearing MOPC-315 had a very high initial count of spleen lymphocytes, which did not further increase upon immune stimulation.**

**Incubation of lymphocytes from plasmacytoma-bearing mice with PHA did not produce an increase in TdR incorporation and in some cases even caused a decrease in TdR incorporation.**

**Lymphocytes from mice bearing TEPC-183, SPQC-11, and MOPC-315 incorporated less TdR in response to LPS than did normal mice. On the other hand, mice bearing MPC-11 incorporated about as much TdR as did normal mice following LPS stimulation. Thus, the defect in the ability to respond to LPS *in vitro* correlated with the lack of an increase of spleen lymphocytes in mice bearing these tumours following antigenic stimulation *in vivo*.**

**No immunodepressive properties of serum from mice with plasmacytoma could be detected.**

MULTIPLE myeloma, both in man (Fahey *et al.*, 1963; Cone and Uhr, 1964; Dammaco and Clausen, 1966) and the mouse (Smith *et al.*, 1960; Fahey and Humphrey, 1962; Hirano *et al.*, 1968; Zolla *et al.*, 1974) often produces a marked humoral immunodepression. Associated with this depressed antibody response is the tendency of patients with myeloma to fall victim to infectious diseases (Fahey *et al.*, 1963; Zinneman and Wall, 1964; Scharff and Uhr, 1965; Meyers *et al.*, 1972). The mechanism of this humoral immunodepression is the subject of considerable controversy.

Hypotheses advanced to explain the humoral immunodepression of myeloma fall into two broad classes: those attributing it to a circulating immunodepressive factor, and those invoking a defect in the antibody-producing cells themselves. Proponents of the existence of circulating immunodepressive factors have suggested that these may be viral RNA (Heller *et al.*, 1973; Chen *et al.*, 1975) or a chalone-like substance (Tanapatchaiyapong and Zolla, 1974). The role of the myeloma protein in the causation of this humoral immunodepression is questionable, since there is no correlation between the type or quantity of the monoclonal protein and the impairment of the antibody response (Fahey *et al.*, 1963). Plasmacytomas that do not secrete any monoclonal immunoglobulins can also cause humoral immunodepression (Zolla, 1972). Other authors have presented evidence suggesting a defect at the cellular level (Jones and McFarlane, 1975; Padarathsingh *et al.*, 1976). Our preliminary studies have revealed that, unlike normal mice, those bearing certain plasmacytomas do not respond to antigenic stimulation with an increase in spleen lymphocytes (Brus *et al.*, 1975). This phenomenon could be due to an impaired recall of lymphocytes to the spleen, or alternatively, an inability of lymphocytes to proliferate in response to stimulation.

The present studies were undertaken to determine whether the lack of increase in spleen lymphocytes following immunization with sheep red blood cells (SRBC) is related to an inability to respond to mitogenic stimulation. This was evaluated using mitogens specific for B- and T-cell subpopulations. The results presented here indicate a cellular defect in the lymphocytes of plasmacytoma-bearing mice.

### MATERIALS AND METHODS

*Tumours.*—The following mouse myelomas, all of BALB/c origin, were used: TEPC-183 and SPQC-11, both of which cause an impaired antibody response, and MOPC-315 and MPC-11, which do not cause humoral immunodepression. TEPC-183 produces an IgM (k) protein, SPQC-11 and MPC-11 both produce an $IgG_{2b}$ (k) protein, and MOPC-315 produces an IgA (k) protein. Immunoglobulin production of these tumours was monitored by immunoelectrophoresis. These tumours were maintained by s.c. inoculation of 0·15 ml of tumour-cell suspension into BALB/c female mice. TEPC-183 and SPQC-11 were kindly given to us by Dr Susan Zolla-Pazner of the V. A. Hospital, New York University Medical School; MOPC-315 was given to us by Dr Herman Eisen of the Massachusetts Institute of Technology; and MPC-11 was a gift from Dr Matthew Scharff of the Albert Einstein School of Medicine.

*Serum.*—Blood was collected from the axillary artery. After clotting, the serum was collected by centrifugation and used immediately.

*Immunization.*—Mice were immunized with i.p. injection of 0·15 ml of 25% SRBC, 18–21 days after tumour inoculation. Control mice were given equivalent volumes of sterile saline. Four and one-half days after immunization, at the peak of the primary immune response, serum was collected for haemolysin determination. At this time the tumours ranged from 10 to 25% of total body weight. Tumours outside this range were not included in this study. The haemolysins were determined by a microtitre technique in which serial 2-fold dilutions of serum, heated at 56°C for 20 min, were made in 0·25 ml of 0·9% NaCl solution at initial dilution of 1 : 1. After addition of equal volumes of 1·5% SRBC and guinea-pig complement diluted 1 : 5 with 0·9% saline, the mixtures were incubated at 37°C for 30 min and then at 4°C overnight. The highest dilution showing complete haemolysis was used as the haemolysin titre.

*Total spleen lymphocyte evaluation.*—Spleens from normal BALB/c mice and plasmacytoma-bearing mice were removed and weighed. The cells were then gently squeezed out of the spleen capsule with a plastic syringe plunger into a sterile cold RPMI-1640 medium supplemented with penicillin (100 μg/ml), streptomycin (50 μg/ml) and glutamine (3 μM/ml). The cells were passed several times through a 20-gauge needle to eliminate clumps, and washed with cold medium. The spleen-cell suspension was layered on a Hypaque–Ficoll gradient (1·078 density) and centrifuged for 40 min at room temperature at 400 *g*. Cells were removed from the interphase with a Pasteur pipette and washed twice with medium at 100 *g* for 10 min. Smears of these cells, stained with Wright's stain, showed that 94–98% of this population were morphologically identifiable as lymphocytes. The remaining fraction of cells were monocytes. Similar results were obtained by examination of these cells by phase microscopy, where lymphocytes were differentiated from monocytes by the ability of the latter to ingest latex particles. The lymphocyte count and viability were determined on a Biophysics Cytograf 6300A and expressed as total lymphocyte count per spleen (TLC/S). The viability ranged from 89 to 96%. Imprints and frozen sections of the spleens were taken and stained with Wright's or haematoxylin-eosin (HE) stain, respectively.

*Mitogenic stimulation.*—Lymphocytes were prepared as previously described. Pooled lymphocytes isolated from 5–8 spleens in each group were used. All cultures were set up in triplicate in flat-bottomed microplates (3040 Microtest II, Falcon Plastics). The final culture volume was 0·2 ml containing $10^6$ lymphocytes per well. PHA-P was obtained from Difco Laboratories, Detroit, Mich. (48232). The final concentration of PHA-P was 5 $\mu$g/well. This dose gave optimal stimulation as determined by a dose-response curve.

Lipopolysaccharide LPS (*E. coli* 055: $B_5$) from Difco Laboratories was used in a final concentration of 30 $\mu$g/well, this dose gave the optimal stimulation.

All wells containing PHA-P were supplemented with 5% fresh, decomplemented Millipore-filtered pooled mouse serum. The optimal LPS response was obtained without addition of serum. Serum was only used with this mitogen when comparing the effect of serum from normal and plasmacytoma-bearing mice.

Microplates were incubated for 48 h at 37°C in 5% $CO_2$ and 95% air. Four hours before incubation was terminated, the cells were pulsed with 1 $\mu$C of $^3$H-TdR (methyl $^3$H) from Schwarz/Mann, Orangeburg, N.Y., sp. act. 1·9 Ci/mM. Cells were harvested in a MASH harvester (Rockefeller University Workshop). The filter papers on which the cells were harvested were dried, placed in vials with Bray's scintillation fluid and counted in a Packard liquid scintillation counter. Results are expressed both as the mean of ct/min of triplicate cultures±s.e. mean and as stimulation index (ct/min stimulated/ct/min unstimulated).

*Statistical analysis.*—Statistical analysis was performed using Student's *t* test.

### RESULTS

### *Effect of mouse plasmacytomao n spleen lymphocytes*

After immunization, non-tumour-bearing mice showed >100% increase in TLC/S compared to mice injected with saline only (Table I). The haemolysin titres of immunized normal mice were always in the range of 6–10 (log 2). Mice bearing plasmacytomas TEPC-183 and SPQC-11 show a markedly impaired antibody response. Associated with this immunodepression is a lack of an increase in TLC/S following antigen stimulation.

Mice bearing tumour MPC-11, which does not interfere with the antibody response, showed a marked increase in TLC/S following immunization.

Mice bearing the plasmacytoma MOPC-315 demonstrate a normal or sometimes

TABLE I.—*Effect of Immunization with Sheep Red Blood Cells (SRBC) on Total Lymphocyte Counts/Spleen (TLC/S) and Haemolysin Titres**

| | Tumour | Treatment | Number of mice | TLC/S × $10^{-6}$ mean ± s.e. | *P* | Haemolysin titre ($\log_2$) |
|---|---|---|---|---|---|---|
| I | None | Saline | 8 | 5·8±1·1 | | 0 |
| | | SRBC | 8 | 16·3±2·7 | <0·005 | 8·9±0·6 |
| | TEPC-183 | Saline | 10 | 3·4±0·6 | | 0 |
| | | SRBC | 11 | 5·8±0·9 | N.S. | 4·6±0·3 |
| II | None | Saline | 7 | 7·5±1·3 | | 0 |
| | | SRBC | 6 | 16·1±3·7 | <0·05 | 7·0±0·3 |
| | SPQC-11 | Saline | 4 | 5·1±2·2 | | 0 |
| | | SRBC | 7 | 3·2±0·9 | N.S. | 4·6±0·3 |
| III | None | Saline | 4 | 9·2±1·0 | | 0 |
| | | SRBC | 4 | 21·2±3·7 | <0·025 | 8·5±1·5 |
| | MPC-11 | Saline | 8 | 17·8±2·7 | | 0 |
| | | SRBC | 8 | 44·8±9·4 | <0·025 | 7·5±0·5 |
| IV | None | Saline | 6 | 7·9±2·4 | | 0 |
| | | SRBC | 6 | 16·0±2·6 | <0·05 | 6·8±1·1 |
| | MOPC-315 | Saline | 8 | 28·6±8·7 | | 0 |
| | | SRBC | 9 | 26·5±6·4 | N.S. | 6·8±0·7 |

* TLC/S were determined 4 days after i.p. injection of 0·15 ml of 0·5% SRBC. Immunizations were done 18–21 days after s.c. tumour inoculation. Lymphocytes were isolated by Ficoll–Hypaque density centrifugation and quantitated with a Biophysics Cytograf 6300 A as described in Materials and Methods.

Haemolysin titres were determined as described in Materials and Methods.

TABLE II.—*PHA Stimulation of Lymphocytes from Normal and Plasmacytoma-bearing Mice*

| Lymphocytes from BALB/c mice bearing: | ct/min × $10^{-3}$* Unstimulated (Mean ± s.e.) | ct/min × $10^{-3}$* Stimulated (Mean ± s.e.) | Stimulation index: ct/min stimulated/ct/min unstimulated Mean ± s.e. |
|---|---|---|---|
| No tumour | 0·81 ± 0·47 | 7·33 ± 3·52 | 10·17 ± 3·49 |
| MOPC-315 | 3·34 ± 1·11 | 1·66 ± 1·16 | 0·67 ± 0·48 |
| No tumour | 0·97 ± 0·58 | 10·25 ± 3·10 | 16·52 ± 3·43 |
| MPC-11 | 4·76 ± 1·81 | 7·93 ± 4·75 | 1·30 ± 0·58 |
| No tumour | 0·32 ± 0·14 | 4·22 ± 2·97 | 9·67 ± 2·86 |
| SPQC-11 | 4·06 ± 1·28 | 0·97 ± 0·82 | 0·20 ± 0·11 |
| No tumour | 0·39 ± 0·15 | 4·91 ± 2·78 | 11·37 ± 2·39 |
| TEPC-183 | 2·56 ± 1·43 | 3·01 ± 2·11 | 0·74 ± 0·27 |

* $10^6$ lymphocytes were incubated in 0·2 ml of RPMI 1640 supplemented with 5% decomplemented normal mouse serum for 48 h at 37°C in an atmosphere of 5% $CO_2$ and 95% air. Cells were stimulated with 5 μg PHA/0·2 ml/well. 4 h before the termination of incubation, the cells were pulsed with $^3$H-TdR. Cells for each experiment were from a pool of lymphocytes derived from 5–8 mice. Each experiment consisted of 3 replicate cultures for each condition. Figures quoted are the mean from 4 experiments.

TABLE III.—*LPS Stimulation of Lymphocytes from Normal and Plasmacytoma-bearing Mice*

| Lymphocytes from BALB/c mice bearing: | No. of experiments | ct/min × $10^{-3}$ Unstimulated (Mean ± s.e.) | ct/min × $10^{-3}$ Stimulated (Mean ± s.e.) | Stimulation index: ct/min stimulated/ct/min unstimulated Mean ± s.e. |
|---|---|---|---|---|
| No tumour | 2 | 0·51 ± 0·29 | 8·15 ± 4·48 | 16·00 ± 0·20 |
| MOPC-315 | | 2·07 ± 0·31 | 5·45 ± 3·88 | 2·97 ± 2·32 |
| No tumour | 8 | 1·13 ± 0·34 | 14·07 ± 2·24 | 16·98 ± 2·93 |
| MPC-11 | | 3·08 ± 0·80 | 15·77 ± 2·48 | 7·08 ± 1·40 |
| No tumour | 5 | 1·36 ± 0·42 | 13·86 ± 3·42 | 11·45 ± 2·27 |
| SPQC-11 | | 2·34 ± 0·67 | 6·48 ± 2·72 | 3·08 ± 1·32 |
| No tumour | 6 | 0·84 ± 0·40 | 11·77 ± 3·74 | 17·59 ± 3·85 |
| TEPC-183 | | 1·36 ± 0·60 | 5·69 ± 1·76 | 6·42 ± 1·21 |

Experiments were performed as described for Table II except that serum was not used. LPS was added to a concentration of 30 μg/0·2 ml/well.

even greater than normal antibody response. Spleens from mice bearing this tumour have an unusually high number of lymphocytes before immunization. Injection of SRBC does not further elevate the TLC/S in this tumour.

Mice bearing plasmacytomas had markedly enlarged spleens, ranging from 160 to 690 mg. Normal mouse spleens averaged 110 mg. However, there was no correlation between spleen lymphocyte count and spleen weight. No metastatic tumour was seen in the spleens upon macroscopic examination. Imprints and frozen sections of spleens from all plasmacytoma-bearing mice showed the presence of some tumour cells. However, the number of tumour cells present was negligible compared to the total lymphocytes. The splenomegaly of tumour-bearing mice correlated with an increase in total leucocytes, erythrocytes and platelets in their spleens.

*PHA stimulation of lymphocytes*

Lymphocytes from all plasmacytoma-bearing mice showed a markedly impaired ability to respond to PHA relative to those from normal mice. As shown in Table II, the net amount of TdR incorporated by lymphocytes from tumour-bearing mice was less than that of cells from normal mice. Lymphocytes from both MOPC-315 and SPQC-11 showed an actual decrease in TdR incorporation relative to non-stimulated lymphocytes from mice bearing these tumours. Lymphocytes from mice with TEPC-183 showed no difference between stimulated and unstimulated

TABLE IV.—*Effect of Serum from Normal and Plasmacytoma-bearing Mice on PHA Stimulation of Lymphocytes from BALB/c Mice*

| Serum from mice bearing: | No. of experiments | ct/min × $10^{-3}$ Unstimulated Mean ± s.e. | ct/min × $10^{-3}$ Stimulated Mean ± s.e. | Stimulation index: ct/min stimulated/ct/min unstimulated Mean ± s.e. |
|---|---|---|---|---|
| No tumour | 10 | 1·00±0·37 | 11·16±3·45 | 11·87±1·93 |
| MOPC-315 | | 1·13±0·34 | 11·78±3·36 | 10·74±2·21 |
| No tumour | 5 | 0·86±0·46 | 9·41±2·54 | 16·06±2·70 |
| MPC-11 | | 0·76±0·36 | 10·52±3·36 | 18·74±3·26 |
| No tumour | 6 | 0·35±0·09 | 4·33±1·92 | 10·17±2·00 |
| SPQC-11 | | 0·28±0·08 | 2·63±1·22 | 8·72±1·93 |
| No tumour | 9 | 0·44±0·14 | 7·12±2·79 | 12·38±1·93 |
| TEPC-183 | | 0·82±0·49 | 8·17±4·21 | 8·67±2·97 |

The experiments were performed as described for Table II. The cells were incubated with either 5% normal mouse serum or 5% serum from tumour-bearing mice.

TABLE V.—*Effect of Serum from Normal and Plasmacytoma-bearing Mice on LPS Stimulation of Lymphocytes from BALB/c Mice*

| Serum from mice bearing: | No. of experiments | ct/min × $10^{-3}$ Unstimulated Mean ± s.e. | ct/min × $10^{-3}$ Stimulated Mean ± s.e. | Stimulation index: ct/min stimulated/ct/min unstimulated Mean ± s.e. |
|---|---|---|---|---|
| No tumour | 3 | 2·21±0·93 | 9·59±3·33 | 5·04±0·98 |
| MOPC-315 | | 1·73±0·52 | 12·36±3·02 | 7·72±1·12 |
| No tumour | 8 | 1·41±0·42 | 5·35±1·33 | 4·19±0·62 |
| MPC-11 | | 1·17±0·31 | 7·82±2·31 | 6·22±0·56 |
| No tumour | 4 | 1·55±0·43 | 6·51±2·09 | 4·08±0·53 |
| SPQC-11 | | 0·88±0·09 | 5·74±1·75 | 6·28±1·46 |
| No tumour | 6 | 1·28±0·50 | 5·12±2·00 | 4·06±0·46 |
| TEPC-183 | | 1·08±0·52 | 6·78±2·60 | 7·69±1·66 |

These experiments were performed as described for Table II except that 5% normal mouse serum or 5% serum from tumour-bearing mice was used.

counts. Lymphocytes from mice bearing MPC-11 had lower net TdR incorporation than did lymphocytes from non-tumour-bearing mice.

*LPS stimulation of lymphocytes*

When stimulated with LPS, lymphocytes from mice bearing MOPC-315, SPQC-11 and TEPC-183 had a lower net TdR incorporation than did those from normal mice. However, lymphocytes from MPC-11-bearing mice incorporated the same net amount of TdR as did lymphocytes from normal mice.

As shown in Tables II and III, all unstimulated lymphocytes from plasmacytoma-bearing mice showed higher basal TdR incorporation than did lymphocytes from normal BALB/c mice. It was noted that unusually high unstimulated counts were seen in 3 experiments where lymphocytes contained much greater than normal contamination of MOPC-315 tumour cells (not shown in tables). For example, lymphocytes from mice bearing MOPC-315 with greater than usual contamination of tumour cells in the spleen showed $10^5$ counts per well in the absence of mitogen.

*Effect of serum on the mitogenic response of lymphocytes*

Tables IV and V show that incubation of lymphocytes from normal BALB/c mice with serum from plasmacytoma-bearing mice did not impair the response of lymphocytes to either PHA or LPS. In fact, sera from plasmacytoma-bearing mice caused a slight but nonsignificant increase of LPS stimulation, as evidenced by the net increase of TdR incorporated.

### DISCUSSION

Mice bearing plasmacytomas and patients with multiple myeloma often show an impairment of antibody production after antigenic stimulation (Marks, 1953; Lawson *et al.*, 1955). Several studies have shown that mice bearing the plasmacytomas TEPC-183 and SPQC-11 have a markedly impaired primary response, as shown by decreased number of plaque-forming cells after immunization with SRBC (Zolla, 1972; Zolla *et al.*, 1974).

In our own studies, normal mice show an increase of both TLC/S and antibody titres after SRBC injection (Table I). However, the plasmacytomas TEPC-183 and SPQC-11 cause a depressed antibody response and lack of spleen lymphocyte increase after SRBC immunization. As shown in Table I, plasmacytomas MOPC-315 and MPC-11 do not affect the antibody response. Mice bearing MPC-11 show lymphocyte counts similar to that of normal mice after SRBC immunization. However, in mice bearing plasmacytoma MOPC-315, stimulation does not result in further increase of their already high unstimulated lymphocyte counts.

In order to determine whether the lack of an increase of spleen lymphocytes following immunization of mice bearing the humorally immunodepressive plasmacytomas is due to inability to proliferate when stimulated, we evaluated their response to mitogens specific for B- and T-cell subpopulations. Mitogenic response can be evaluated by stimulation index, total number of counts incorporated, or the net increase in the number of counts following mitogenic stimulation. It is important to stress that stimulation indices are only useful when comparing the stimulation of similar populations of cells. Under these conditions, the denominator (ct/min unstimulated) remains constant, and the only variable is the numerator (ct/min stimulated). However, in our studies, marked differences in the TdR incorporation of unstimulated cultures make the stimulation index an ambiguous parameter. A more useful parameter is the *net* increase (ct/min stimulated—ct/min unstimulated) in TdR incorporation following mitogenic stimulation. While our results (Tables II–V) are given as all 3 of the parameters listed above, interpretations were made using the net increase in counts.

PHA is known to be a T-cell mitogen in mice (Dukor and Dietrich, 1967; Doenhoff *et al.*, 1970; Andersson and Blomgren, 1971; Blomgren and Svermyr, 1971). Our data on the effect of PHA on lymphocytes of plasmacytoma-bearing mice show a marked decrease in net TdR incorporated following mitogenic stimulation. Studies by Zolla-Pazner, Sullivan and Richardson (1976) on lymph-node cells from plasmacytoma-bearing mice, show that the response of these cells to PHA does not differ dramatically from that of normal mice. However, their data on spleen cells from one line of plasmacytoma-bearing mice revealed an impairment of the response to PHA similar to those repeated here by us. These studies are in agreement with the preliminary data on mitogenic response of spleen cells from plasmacytoma-bearing mice as reported by Padarathsingh *et al.* (1976). Peripheral lymphocytes from patients with multiple myeloma also show a depressed response to PHA (Jones and MacFarlane, 1975). It should be noted that the impaired response to PHA may not be related to the humoral immunodepression associated with myelomas, since our data indicate that the response to this mitogen was depressed even in the lymphocytes from plasmacytoma-bearing mice capable of a normal antibody response to SRBC, a T-cell-dependent antigen.

B-lymphocyte function was evaluated with LPS, a B-cell mitogen (Peavy *et al.*, 1970; Gery *et al.*, 1972). The results of LPS stimulation of lymphocytes correlate with the data presented on lymphocyte proliferation after immunization with SRBC. Immunization of mice bearing MOPC-315, TEPC-183, and SPQC-11 does not result in an increase in total spleen lymphocytes, nor do mice bearing these tumours respond normally to LPS stimulation. Lymphocytes from mice bearing MPC-11, which had a significant increase in total spleen lymphocytes following immunization, show normal amounts of TdR incorporated after LPS stimulation (Tables I and III). The presence of some tumour cells in the preparation of lymphocytes from plasmacytoma-bearing mice may be related to the high basal TdR incorporation of unstimulated cultures (Tables II and III). However, it is unlikely that this elevated TdR incorporation in unstimulated cultures from plasmacytoma-bearing animals is due to the metabolism of tumour cells, since spleen imprints show only a small proportion of the cells to be morphologically identifiable as tumour cells. Nevertheless, these malignant plasma cells may serve as a constant antigenic stimulus to the lymphocytes. It has been shown that *in vivo* administration of antigen to normal mice causes transient inhibition of the PHA response of their spleen cells *in vitro* (Gershon *et al.*, 1974). We are now studying the possible role of metastatic tumour cells in the impairment of lymphocytes after mitogenic or antigenic stimulation.

Sera from plasmacytoma-bearing mice do not adversely affect the mitogen response of lymphocytes to both mitogens (Table IV and V). The presence of humoral immunosuppressive factors in multiple myeloma has been proposed (Zolla, 1972; Zolla *et al.*, 1974); Tanapatchaiyapong and Zolla, 1974). However, we could find no evidence of suppressive factor(s) in our system. Thus, if such a factor exists in serum from plasmacytoma-bearing mice, it may be very unstable or present in ineffective concentrations in the experimental system used. We could not evaluate the effect of higher serum concentrations, as these proved to be inhibitory to our cultures. However, our results presented here on the mitogenic response of lymphocytes from plasmacytoma-bearing mice suggest a cellular site of the defect in plasmacytoma.

At present, we are continuing our search for immunosuppressive factors in the serum, using diffusion chambers *in vivo* and serum fractions *in vitro*. We are evaluating the response of lymphocytes from plasmacytoma-bearing animals to antigenic stimulation *in vitro*, using defined lymphocyte and monocyte populations.

This investigation was supported by Grant Numbers P30 14194 and CA 12635, awarded by the National Cancer Institute, DHEW.

The authors wish to thank Mr Steven Reisman and Mr Steven Bodine for their excellent technical assistance.

### REFERENCES

Andersson, B. & Blomgren, H. (1971) Evidence for Thymus Independent Humoral Antibody Production in Mice against Polyvinylpyrrolidone and *E. coli* Lipopolysaccharide. *Cell. Immun.*, **2**, 411.

Blomgren, H. & Svermyr, E. (1971) Evidence for Thymic Dependence of PHA Reactive Cells in Spleen and Lymph Nodes and Independence in Bone Marrow. *J. Immun.*, **106**, 235.

Brus, I., Brent, J. & Hollander, V. P. (1975) Effect of Mouse Plasmacytomas on Spleen Lymphocyte Content. 3rd Meeting, *Int. Soc. of Haemat.*, London.

Chen, Y., Bhoopalam, N., Yakulis, V. & Heller, P. (1975) Changes in Lymphocyte Surface Immunoglobulins in Myeloma and the Effect of an RNA-containing Plasma Factor. *Ann. intern. Med.*, **83**, 625.

Cone, L. & Uhr, J. W. (1964) Immunological Deficiency Disorders Associated with Chronic Lymphocytic Leukemia and Multiple Myeloma. *J. clin. Invest.*, **43**, 2241.

Dammaco, F. & Clausen, J. (1966) Antibody Deficiency in Paraproteinemia. *Acta. med. scand.*, **179**, 755.

Doenhoff, M. J., Davies, A. J. S., Leuchars, E. & Wallis, V. (1970) The Thymus and Circulating Lymphocytes of Mice. *Proc. R. Soc. Lond., B.*, **176**, 69.

Dukor, P. & Dietrich, F. M. (1967) Impairment of Phytohemagglutinin induced Blastic Transformation in Lymph Nodes from Thymectomized Mice. *Int. Archs Allergy*, **32**, 521.

Fahey, J. L., Scoggins, R., Utz, J. P. & Szwed, C. F. (1963) Infection, Antibody Response and Gamma Globulin Components in Multiple Myeloma and Macroglobulinemia. *Am. J. Med.*, **35**, 648.

Fahey, J. L. & Humphrey, J. H. (1962) Effect of Transplantable Plasma Cell Tumors on Antibody Response in Mice. *Immunology*, **5**, 110.

Gershon, R. K., Gery, I. & Waksman, B. H. (1974) Suppressive Effects of *in vivo* Immunization on PHA Responses *in vitro*. *J. Immun.*, **112**, 215.

Gery, I., Kruger, J. & Spiesel, S. Z. (1972) Stimulation of B-lymphocytes by Endotoxin. Reactions of Thymus-deprived Mice and Karyotypic Analysis of Dividing Cells in Mice Bearing $T_6T_6$ Thymus Grafts. *J. Immun.*, **108**, 1088.

Heller, P., Bhoopalam, V., Cabana, N., Costea, H. & Yakulis, V. (1973) The Role of RNA in the Immunological Deficiency of Plasmacytoma. *Ann. N.Y. Acad. Sci.*, **207**, 468.

Hirano, S., Imamaura, Y., Takaku, F. & Nakao, K. (1968) Immune Response in Mice with Plasma

Cell Tumor Assayed by Agar Plaque Technique. *Blood*, **31**, 252.

Jones, S. V. & McFarlane, H. (1975) T and B Cells in Myelomatosis. *Br. J. Haematol.*, **31**, 545.

Lawson, H. A., Stewart, C. A., Paull, A. M. & Phillips, R. W. (1955) Observations on the Antibody Content of Blood in Patients with Multiple Myeloma. *New Engl. J. Med.*, **252**, 13.

Marks, J. (1953) Antibody Formation in Myelomatosis. *J. Clin. Pathol.*, **6**, 62.

Meyers, B. R., Hirshman, S. Z. & Axelrod, J. A. (1972) Current Patterns of Infection in Multiple Myeloma. *Am. J. Med.*, **52**, 86.

Padarathsingh, M., McCoy, J., Dean, J., Lewis, D., Northing, J. & Halper, J. (1976) General and Specific Cell-mediated Immunocompetence of BALB/c Mice Bearing a Chemically Induced Plasmacytoma. *Fed. Proc.*, **35**, 387.

Peavy, D. L., Adler, W. H. & Smith, R. T. (1970) The Mitogenic Effects of Endotoxin and Staphylococcal Enterotoxin B on Mouse Spleen Cells and Human Peripheral Lymphocytes. *J. Immun.*, **105**, 1453.

Scharff, M. D. & Uhr, J. W. (1965) Immune Deficiency Disorders Associated with Lymphoproliferative Diseases. *Sem. Haemat.*, **2**, 47.

Smith, F., Grenan, M. M. & Owens, J. (1960) Effects of a Transplanted Plasma Cell Tumor on Antibody Formation. *J. natn. Cancer Inst.*, **25**, 803.

Tanapatchaiyapong, P. & Zolla, S. (1974) Humoral Immunosuppressive substance in Mice Bearing Plasmacytomas. *Science, N.Y.*, **186**, 748.

Zinneman, H. H. & Wall, W. H. (1964) Recurrent Pneumonia in Multiple Myeloma and Some Observations of Immunologic Response. *Ann. intern. Med.*, **41**, 1152.

Zolla, S. (1972) The Effect of Plasmacytomas on the Immune Response of Mice. *J. Immun.*, **108**, 1039.

Zolla, S., Naor, D. & Tanapatchaiyapong, P. (1974) Cellular Basis of Immunodepression in Mice with Plasmacytomas. *J. Immun.*, **112**, 2068.

Zolla-Pazner, S., Sullivan, B. & Richardson, D. (1976) Cellular Specificity of Plasmacytoma-induced Immunosuppression. *J. Immun.*, **117**, 563.